# Reading fluency during the COVID-19 pandemic: a longitudinal and cross-sectional analysis

**DOI:** 10.1055/s-0042-1758446

**Published:** 2022-12-19

**Authors:** Luciana Mendonça Alves, Isa Mourão Carvalho, Luiz Felipe dos Santos, Gabriela de Lima Ribeiro, Laura de Souza Cardoso Freire, Vanessa de Oliveira Martins-Reis, Ludimila Labanca, Letícia Correa Celeste

**Affiliations:** 1 Universidade Federal de Minas Gerais, Faculdade de Medicina, Departamento de Fonoaudiologia, Belo Horizonte MG, Brazil.; 2 Universidade Federal de Minas Gerais, Faculdade de Medicina, Programa de Pós-graduação em Ciências Fonoaudiológicas, Belo Horizonte MG, Brazil.; 3 Universidade de Brasília, Faculdade de Ceilândia, Curso de Fonoaudiologia, Brasília DF, Brazil.; 4 Universidade de Brasília, Faculdade de Ceilândia, Programa de Pós-graduação em Ciências da Reabilitação, Brasília DF, Brazil.

**Keywords:** Reading, Education, COVID-19, Learning, Primary Education, Leitura, Educação, COVID-19, Aprendizagem, Ensino Fundamental I

## Abstract

**Background** International studies performed during the periods of social isolation highlighted the potential loss of student's learning skills. The present study fills a gap in Brazilian research on this topic and focuses on the development of reading fluency.

**Objective** To investigate the development of the reading fluency of students in the early years of elementary school during e-learning as a result of the social distancing measures put into effect due to the coronavirus disease 2019 (COVID-19) pandemic.

**Methods** Students from grades 2 to 5 were recorded. The number of words read per minute and of those read correctly per minute were analyzed. Descriptive statistical analysis was performed, using analysis of variance (ANOVA) for repeated measures with Bonferroni correction in the longitudinal study, and the *t* -test in the cross-sectional study.

**Results** In the cross-sectional study, 162 students participated. Only the comparison between the 2nd grade classes of 2020 and 2021 showed a statistically significant difference. In the prepandemic classes, the students had better results in reading accuracy than the students assessed during the pandemic. The longitudinal study included 75 students, who improved in fluency rate and accuracy as expected between March and December 2020. In March 2021, the results showed a drop, which may be related to school closures during the Brazilian summer vacation.

**Conclusions** The present research demonstrates the results of Brazilian students in terms of the development of reading fluency during the pandemic. There was an expressive development in grades 2 and 3, with stability in the following grades. The 2nd grade class of 2021 suffered a major impact due to the pandemic.

## Resumo

**Antecedentes** Estudos internacionais realizados durante os períodos de isolamento social destacaram a potencial perda das habilidades de aprendizagem dos alunos. O presente estudo preenche uma lacuna nas pesquisas brasileiras sobre este tema, e se centra no desenvolvimento da fluência de leitura.

**Objetivo** Investigar o desenvolvimento da fluência de leitura de alunos nos anos iniciais do Ensino Fundamental durante as aulas remotas ministradas em decorrência das medidas de distanciamento social postas em prática por conta da pandemia de doença do coronavírus 2019 ( *coronavirus disease 2019* , COVID-19, em inglês).

**Métodos** Estudantes do 2 ^o^ ao 5 ^o^ anos foram gravados. Foram analisadoso número de palavras lidas por minuto e o de palavras lidas corretamente por minuto. Realizou-se análise estatística descritiva por meio de análise de variância ( *analysis of variance* , ANOVA, em inglês) para medidas repetidas com correção de Bonferroni no estudo longitudinal, e teste T no estudo transversal.

**Resultados** Ao todo, 162 estudantes participaram do estudo transversal. Apenas a comparação entre as turmas de 2 ^o^ ano de 2020 e 2021 apresentou diferença estatisticamente significativa. Nas aulas pré-pandemia, as turmas apresentaram resultados melhores em termos de precisão de leitura do que as turmas avaliadas durante a pandemia. O estudo longitudinal incluiu 75 alunos; entre março e dezembro de 2020, eles melhoraram sua taxa de fluência e acurácia, conforme esperado. Em março de 2021, os resultados mostraram uma queda, o que pode estar relacionado ao fechamento das escolas durante as férias.

**Conclusões** Esta pesquisa demonstra os resultados do desenvolvimento da leitura de uma amostra de estudantes brasileiros durante a pandemia. Houve uma evolução expressiva no 2° e 3° anos, com estabilidade nos anos seguintes. A turma de 2° ano de 2021 sofreu um grande impacto devido à pandemia.

## INTRODUCTION

The measures adopted to help curb the spread of coronavirus disease 2019 (COVID-19) infections had a dramatic impact on economies and health systems worldwide, as well as a detrimental effect on education. The sudden and prolonged closure of schools and the prioritization of e-learning with an interruption in classroom teaching had a negative effect on the academic world, especially for students who were learning reading and reading comprehension. 1 2 3

A United Nations (UN) report 4 highlighted that ∼ 94% of the world's student population was affected by school closures, which totals 1.58 billion students from primary school to higher education. In Latin America and the Caribbean, the prolonged closure of schools affected the reading comprehension of around 77% of elementary school students, a percentage that reached 55% before the pandemic, with greater detriment to student's education, especially those from low-income families. 5 Although the numbers seem very high, this is the reality that Latin American and Caribbean countries have been living with in recent years. The pandemic has exacerbated a problem that was already important to the region.

Even though it is possible to find data in the international literature that enables us to predict the fluency gain in words per minute during schools terms, in Brazil we only have research that shows that gain in terms of school grades, all obtained transversally. Longitudinal studies are essential to measure the gain in reading over the same school year and between grades. Such information enables teachers to monitor their students' reading gain and make the necessary adjustments to improve their performance.

“Learning loss” is the concept that students learnt less during the pandemic than in previous Years. United Nations stimates that at the end of primary education the learining loss effect could increase by more than 20%, and learning deficits would be substantially larger for the most disadvantaged students.

Learning losses were researched and compared in relation to students' academic performance before and after closure of the schools. To predict the impact of social isolation on schooling, Bao et al. 6 calculated that potential learning losses in reading skills (such as phonological awareness, language structures, word decoding and comprehension) of North American children in kindergarten during school closures and without formal education. The resulting model predicted a 66% less-than-expected gain in learning these reading skills compared with rates achieved in years prior to the pandemic, with a greater impact on students unfamiliar with reading habits.

According to Engzell et al., 7 the results of a Dutch national assessment performed in 2020 after the homeschooling period pointed to an average learning loss of 3.16 percentage points of the national average, which is equivalent to 0.08 standard deviations (SDs) when compared with the results of the previous three assessments.

Domingue et al. 8 evaluated the impact of COVID-19 on the development of oral reading fluency in students from grades 1 to 3 in more than 100 United States school districts. The survey showed that students in grades 2 and 3 had a shortfall of ∼ 30% compared with a typical year, and it pointed out that students in low-performance districts experienced major shortfalls in learning.

Soares 9 discusses that reading is not simply reading words; reading with fluency denotes fast and accurate recognition of words and word sets, with appropriate rhythm and intonation.

It is important to understand the students' reading development during the period that the schools were closed due to COVID-19 so the teachers can plan strategies for the school year after that. Measuring and monitoring the development of reading fluency are quick and highly predictive strategies for the development of reading and reading-comprehension skills, and are considered by researchers 8 10 strong indicators of proficient reading.

Some Brazilian researchers 11 12 who studied the impact of e-learning on literacy focused more on how e-learning models required adjustments in pedagogy, syllabi, and learning techniques in order to meet the demands of both teachers and students. However, it also relevant to understand the impact caused by the inequality in access to digital technologies and the conditions under which school activities were performed in the home environment, as well as the impact on the students' mental health. 11 12

Based on the data that has already been published on the effects of the pandemic on the educational systems of other countries, 7 8 11 12 it has become evident that there is a need to monitor the reading skills of Brazilian students to be able to assess the effect of e-learning on the performance of students. Therefore, the aim of the present study is to investigate the development of reading fluency among students from the early grades of elementary school, grades 2 to 5, during the period of e-learning imposed by the social distancing measures put into effect during the COVID-19 pandemic.

## METHODS

The present is an exploratory study with a selected sample that was approved by the Research Ethics Committee of Universidade Federal de Minas Gerais (UFMG), under protocol CAAE 35588820.0.0000.5149 and number 4.453.235. The participants and their legal guardians filled out a free and informed consent Form and a free and informed consent form for children. There are two experiments presented in this paper, a cross-sectional study and a longitudinal study.

The sample of this study included students from the grades to 2 to 5 of an elementary private school in the capital city of Belo Horizonte, Minas Gerais. It's a school in which ∼ 72% of the students have both parents with a college degree and 91% of them have at least one parent with a college degree.

The criteria defined for participation in the research were enrollment in the initial grades of Elementary School (grades 2 to 5) and participation in all stages of testing. The exclusion criteria were: non-completion of the reading task; giving up on participation; students who had help from their legal guardians during reading; phonological exchanges; and foreign students. Students who failed on their grades were also excluded from the research. In the longitudinal study, those students who for some reason did not participate in some stage of data collection were also excluded.

The recordings were made in different situations. The first recordings were made in person in March 2020 at the school, before the start of the pandemic, in a quiet room using a laptop connected to a unidirectional microphone and running the Praat software 13 for speech analysis and synthesis of the recordings, which were read aloud by the students.

The following recordings were made online by video conference, due to the social distancing measures enacted because of the pandemic, in September and December 2020 and March 2021. The parents were asked to choose a quiet place at home for the students to be able to read. The recordings were made outside of regular school hours through Zoom on a computer or on another device with an adequate screen size, to be able to see and read the text.

Prior to the readings, the students were instructed on what to do, and any queries or problems were cleared up; then, the students read aloud the on-screen text. The audio was extracted for analysis of the reading parameters.

The parameters analyzed in the reading recordings were the number of words read per minute (WPM) – the fluency rate – and the number of words read correctly per minute (WCPM) – the accuracy. The data were obtained by listening to the recordings associated with the manual analysis of the parameters, which were updated in an Excel (Microsoft Corp., Redmond, WA, United States) spreadsheet.

Descriptive data analysis was performed using frequency analysis for the categorical variables (sex) and measures of central tendency (mean) and variability (standard deviation) for the continuous variables (WPM and WCPM). The Shapiro-Wilk test was used to analyze if the continuous variables had normal distribution.

### Cross-sectional study

#### *Sample characteristics*

A total of 162 students from the grades 2 to 5 of elementary school were included in the study, 82 girls and 80 boys. In 2020, their average age was: 6.9 years in grade 2; 7.9 years in grade 3; 8.9 years in grade 4; and 10 years in grade 5. In the following year, 2021, the average ages were: 6.8 years in grade 2; 7.9 years in grade 3; 8.9 years in grade 4; and 9.9 years in grade 5.

**Figure 1:**
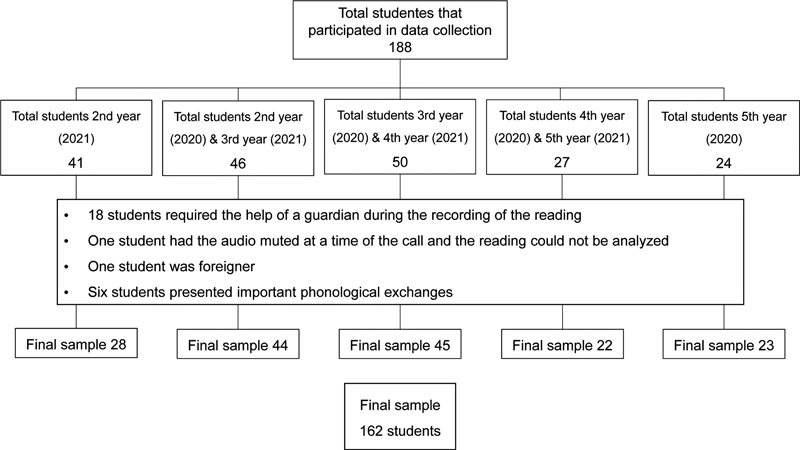
Flowchart of the sample of the cross-sectional study.

Considering that most of the students underwent more than one evaluation, we performed 237 evaluations on 162 students. The illustration of this sample can be found in Figure 1 . In total, 147 students were assessed in March 2020 and 164, in March 2021.

Using the *t* -test, we conducted a comparative analysis of the performances of the students who started grades 2 to 5 during on-site education (prepandemic, in March 2020) and those who started grades 2 to 5 during emergency e-learning (during the pandemic, in March 2021).

### Longitudinal study

#### *Sample characteristics*

**Figure 2:**
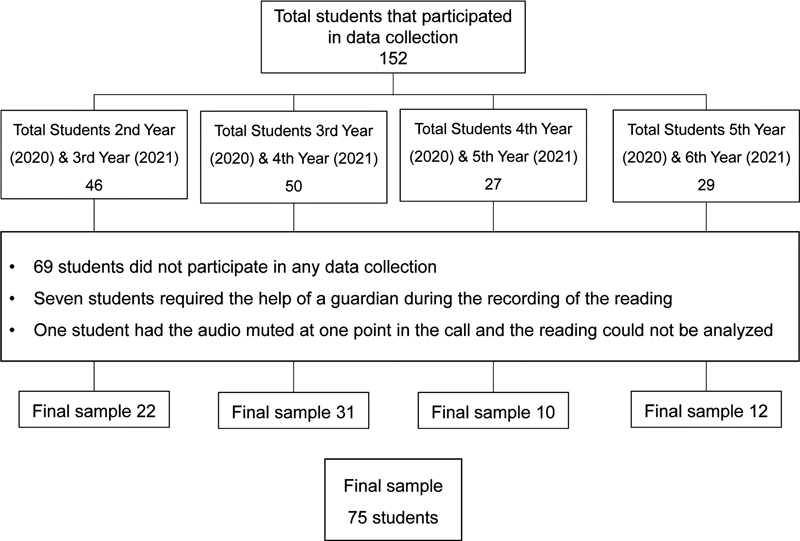
Flowchart of the sample of the longitudinal study.

The longitudinal study included 75 students from grades 2 o 5 of elementary school, 41 boys and 34 girls, who had their reading fluency assessed in March 2020 (in person), September 2020 (online), December 2020 (online), and March 2021 (online). Of these, 22 were from grade 2, 31, from grade 3, 10, from grade 4, and 12 from grade 5. Figure 2 illustrates the sample data.

Texts that were standardized for speakers of Brazilian Portuguese were used for the evaluation of reading fluency. These texts were published for each grade. 14 15 16 To avoid the effect of the previous text training, a different text was used for each evaluation.

We also performed an analysis of the follow-up of the reading fluency performance of the students. The performance of the same students was compared in the months of March, September, and December 2020, and March 2021. In the analysis regarding March 2021, the students were from grades 3 to 6. We used analysis of variance (ANOVA) for repeated measures with Bonferroni correction.

## RESULTS

### Cross-sectional study

#### *Reading performance: WPM and WCPM*

**Figure 3:**
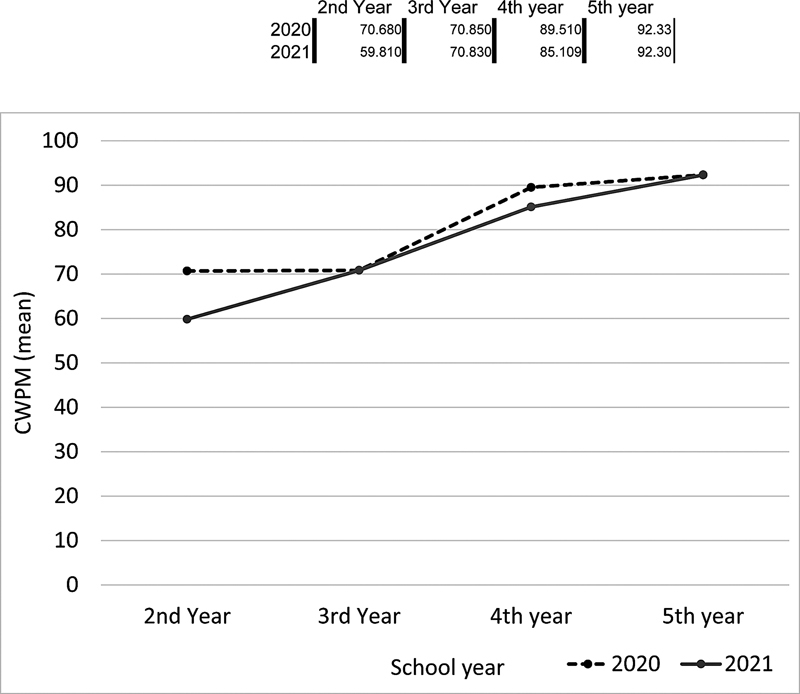
Rate of words read correctly per minute among students from grades 2 to 5 before the COVID-19 pandemic (March 2020) compared with the period during the pandemic (March 2021). Abbreviation: WCPM, words read correctly per minute.

The comparison of the WPM performance of students in the prepandemic (March 2020) and pandemic (March 2021) periods is shown in Table 1 . Table 2 and Figure 3 show the students' WCPM performance. There was a significant statistical difference when comparing the mean WPM and WCPM in grade 2 before and during the pandemic ( *p* < 0.05). The prepandemic performance of students in grade 2 was better than that of those in grade 2 assessed during the pandemic. The comparisons between the following grades were not statistically relevant, indicating that there was no significant difference between the 2020 and 2021 assessments for grades 3 to 5.

**Table 1:** Rate of words read per minute among students in grades 2 to 5 before (March 2020) and during (March 2021) the pandemic

Characteristics	Grade 2		Grade 3		Grade 4		Grade 5	
	March/20	March/21	March/20	March/21	March/20	March/21	March/20	March/21
N	40	28	41	30	22	37	23	16
Average	75.682	59.591	76.097	73.032	93.391	87.656	96.34	98.48
Median	74.755	56.812	75.135	75.262	98.595	86.331	93.00	101.93
Standard deviation	26.922	22.936	22.715	23.624	21.374	21.143	17.69	20.35
Minimum	27.439	15.966	36.483	34.926	47.090	46.901	66.18	57.67
Maximum	144.218	105.179	128.111	122.160	133.120	130.638	134.13	137.84
*p* -value	0.012		0.583		0.583		0.73	

Note: * *t* -test is statistcally significant when *p* -value is lower than 0.05.

**Table 2:** Rate of words read correctly per minute among students in grades 2 to 5 before (March 2020) and during (March 2021) the pandemic

Characteristics	Grade 2		Grade 3		Grade 4		Grade 5	
	March/20	March/21	March/20	March/21	March/20	March/21	March/20	March/21
N	40	28	41	30	22	37	23	16
Average	70.684	56.810	70.856	70.830	89.519	85.109	92.33	92.30
Standard deviation	70.459	55.831	71.892	73.677	95.500	84.481	90.29	95.04
Minimum	28.104	22.834	22.251	23.267	21.849	21.229	17.62	19.95
Maximum	23.434	12.700	33.094	33.691	44.060	45.896	60.07	46.47
*p* -value	140.940	102.789	121.659	120.403	129.320	125.000	128.92	124.67
Average	0.034		0.996		0.996		0.996	

Note: * *t* -test is statistcally significant when *p* -value is lower than 0.05.

The performance of students in grade 2 in 2021 was worse than that of those in the same grade in 2020, with an average difference of 16 words per minute ( *p* < 0.005). Those results show a delay in both the reading rate and accuracy after the schools remained closed for one year, with only remote learning for students in the early years of literacy development. As for the other grades, there was no delay regarding reading rate and accuracy.

### Longitudinal study

Table 3 shows the students' WPM and WCPM performances in March, September, and December 2020, and in March 2021. The results show a tendency of improvement and that there was a statistically significant difference when comparing the performance of students in the periods evaluated.

**Table 3:** Student performance regarding WPM and WCPM in March, September, and December 2020, and in March 2021

Grade	Evaluation period	WPM			WCPM		
		Average	SD	*p* -value	Average	SD	*p* -value
2	March/20	74.41	25.13	< 0.001	69.01	27.47	< 0.001
	September/20	82.06	24.55		78.78	25.02	
	December/20	123.64	34.64		121.04	35.19	
	March/21	75.26	25.33		72.97	24.92	
3	March/20	75.71	23.22	< 0.001	71.13	22.56	< 0.001
	September/20	90.65	26.19		87.55	26.40	
	December/20	102.22	25.32		98.14	24.09	
	March/21	86.92	20.95		84.51	20.95	
4	March/20	92.33	25.65	0.009	88.77	26.16	0.012
	September/20	89.68	24.89		87.56	24.70	
	December/20	109.95	19.94		105.80	18.55	
	March/21	95.57	21.60		89.34	22.46	
5	March/20	103.59	19.08	0.003	100.10	18.62	0.004
	September/20	103.28	14.29		99.87	14.29	
	December/20	125.47	16.73		122.60	17.09	
	March/21	104.41	17.23		100.47	16.10	

Abbreviations: SD, standard deviation; WCPM, words read correctly per minute; WPM, words read per minute.

Note: * Analysis of variance for repeated measures is statistcally significant when *p* -value is lower than 0.05.

Table 4 shows the periods in which statistically significant differences in terms of of WPM and WCPM measurements were observed. Those results indicate that among grade 2 students, there was a significant increase in WPM and WCPM throughout the 2020 tests. In March 2021, the performance of the students suffered a significant decreased, becoming similar to their performance in March 2020. As for students in grades 3 to 5, the decrease was smaller, as they maintained their September 2020 performance.

**Table 4:** Comparisons of student performance regarding WPM and WCPM in March, September, and December 2020, and in March 2021

Grade	Comparisons		WPM: *p* -value	WCPM: *p* -value
2	March/20	September/20	0.023 ^+^	0.005 ^+^
		December/20	0.000 ^+^	0.000 ^+^
		March/21	1.000	1.000
	September/20	December/20	0.000 ^+^	0.000 ^+^
		March/21	0.487	0.701
	December/20	March/21	0.000 ^+^	0.000 ^+^
3	March/20	September/20	0.000 ^+^	0.000 ^+^
		December/20	0.000 ^+^	0.000 ^+^
		March/21	0.000 ^+^	0.000 ^+^
	September/20	December/20	0.000 ^+^	0.001 ^+^
		March/21	0.941	1.000
	December/20	March/21	0.000 ^+^	0.000 ^+^
4	March/20	September/20	1.000	1.000
		December/20	0.003 ^+^	0.005 ^+^
		March/21	1.000	1.000
	September/20	December/20	0.004 ^+^	0.013 ^+^
		March/21	0.742	1.000
	December/20	March/21	0.026 ^+^	0.006 ^+^
5	March/20	September/20	1.000	1.000
		December/20	0.006 ^+^	0.004 ^+^
		March/21	1.000	1.000
	September/20	December/20	0.001 ^+^	0.001 ^+^
		March/21	1.000	1.000
	December/20	March/21	0.002 ^+^	0.001 ^+^

Abbreviations: SD, standard deviation; WCPM, words read correctly per minute; WPM, words read per minute.

Note: * *p* -value was calculated by adjustment for several comparisons: Bonferroni.

**Figure 4:**
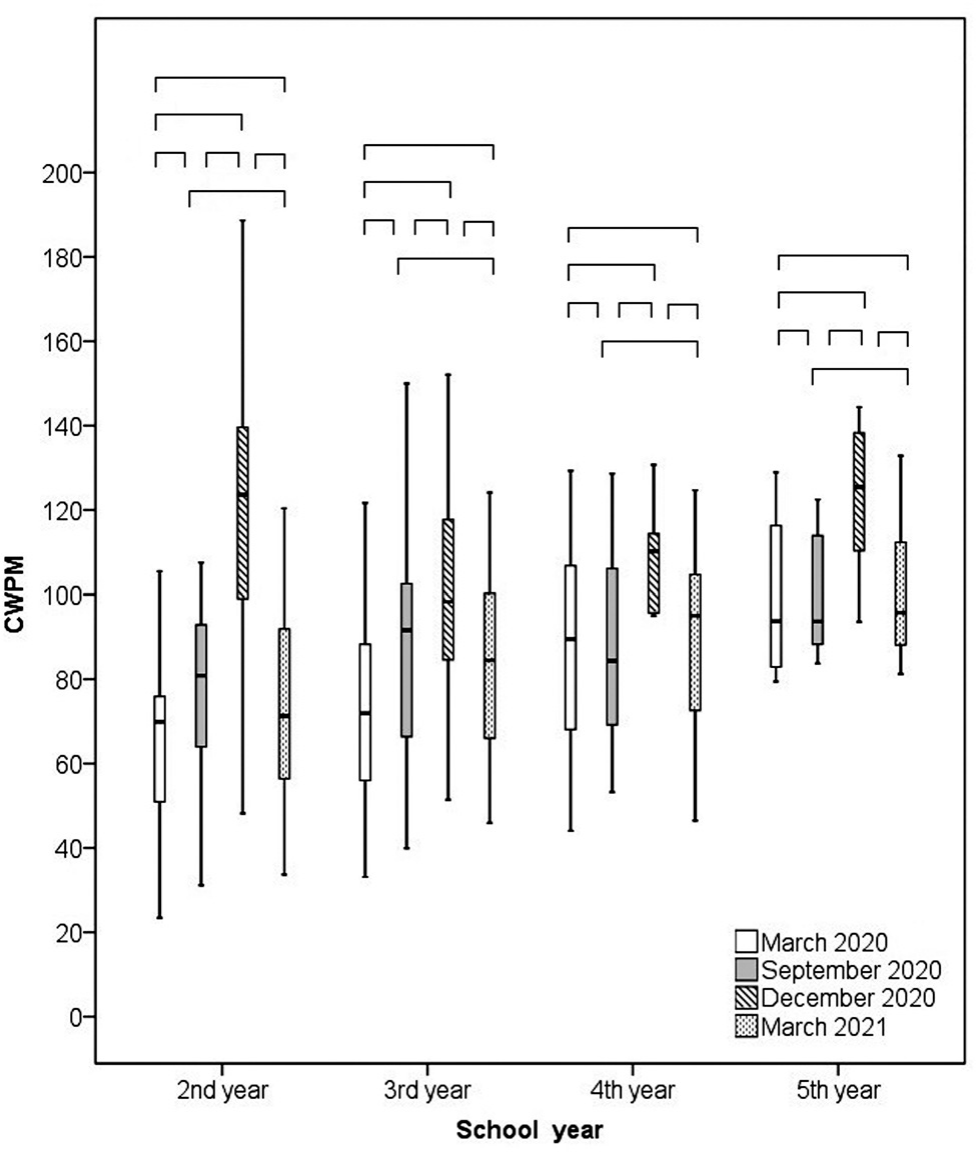
Performance of students from grades 2 to 5 expressed in CWPM in March, September, and December 2020, and in March 2021. Abbreviation: WCPM, words read correctly per minute.

Figure 4 shows the performance of students from grades 2 to 5 regarding the WCPM measurements made in March, September, and December 2020, and in March 2021.

**Figure 5:**
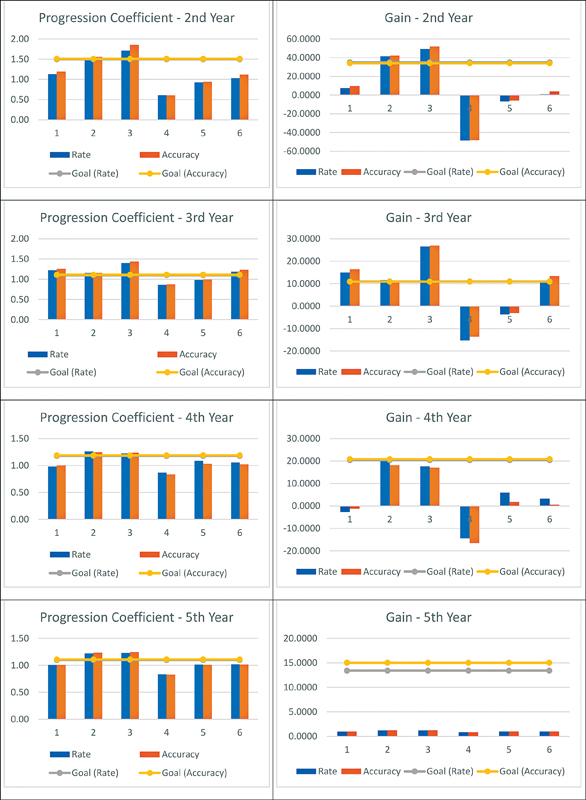
Student's progression coefficient and gain during the assessments.

Figure 5 shows the coefficient of progression 17 and the gain regarding all periods evaluated in the present study compared with the reference values available for Brazilian students in Elementary School. 18 The coefficient of progression was obtained by dividing one performance by the previous one. The gain was obtained by subtracting the previous performance. Thereby, time 1 refers to September 2020 and March 2020; time 2, to December 2020 and September 2020; time 3, to December 2020 and March 2020; time 4, to March 2021 and December 2020; time 5, to March 2021 and September 2020; and time 6 refers to March 2021 and March 2020.

Considering that there is no gain target and coefficient of progression available for Brazilian students, we simulated such targets based on the values available in the cross-sectional study by Alves et al. 18 Therefore, the target earning and coefficient of progression for grade 2 students was calculated using the values available for grade 3 students.

The students reached the goal of the coefficient of progression and gain in time 2. From time 4 onwards, the results were lower than the goal, since students showed decreases in reading rate and accuracy in March 2021 ( Figure 5 ). The graphs also show that the coefficient of progression and gain target are higher for grade 2.

## DISCUSSION

The purpose of the present study is to investigate the development in reading fluency of students in the early years of elementary school during e-learning, which was required due to the social distancing measures enacted during the COVID-19 pandemic. Longitudinal and cross-sectional studies were performed to analyze the development of students throughout 2020 and to compare the performance of students that were evaluated before and during the pandemic using WPM and WCPM.

Table 3 shows that, between March and December 2020, the students improved their reading skills, with an increase in WPM and WCPM, which was to be expected and is in line with the results of other Brazilian studies. 18 19 20 However, in March 2021, the results showed a drop, which may be related to school closures during the school holidays, in which students did not have the same exposure to reading as during the school year.

There was a great improvement in the results of the grade 2 in December 2020. This result may be due to more intensive studies at the end of the year, and it may also have been influenced by the text that was chosen. 21 The text used for the last year 16 was better suited to grade 2. However, the results show that it was apparently easier than the previous ones. This finding, which indicates a possible bias, corroborates what Soares 9 states about the development of reading skills that permeate the careful choice of text.

Statistical significance ( *p* < 0.05) was observed regarding the development of reading in all grades, as reported more accurately in Table 4 . However, grades 2 and 3 stood out, pointing to a constant progression in 2020. In the following grades, there was a greater stability in the comparisons ( Figure 5 ), a phenomenon that is in line with the findings by Alves et al. 18 and the arguments of Soares, 9 Macedo et al. 22 and Salles and Parente, 23 who state that progress in reading skills tends to evolve as the child becomes more exposure to the written word.

Figures 4 and 5 illustrate the aforementioned results, showing an increase in development and moments of stability in 2020 and a drop in results, possibly caused by school closures due to school holidays in 2021, which is known as the summer learning loss phenomenon. 24 The gain rate was greater in grade 2, but from grade 3 on this gain starts to diminish ( Figure 5 ). We must point out here that this rate was calculated based on non-longitudinal studies, since we did not find any reports that provided that data from Brazilian students. Future studies should focus on identifying the expected gain in fluency reading during a school year to guide the school team on decision making.

Alves et al. 18 delimit suggestions of values expected for each grade, as well as the staggered interpretation of the results from a Z score. Compared with these values, the averages found in the present study for the WPM and WCPM ( Table 3 ) are within the expected range, and are not indicative of reading deficits in the assessed students.

In March 2020, grade 3 had a limited value for the standard, as well as grade 5 in September 2020. The values that indicate no deficit are above 71 and above 98 respectively, as the averages found for grade 3 were of 75.71 of WPM and of 71.73 of WCPM, and, for grade 5, a WCPM of 99.87. The findings pertaining to grades 3 and 5 may be closely linked to the effect of the December 2019 and July 2020 school holidays. 24 During this period, students usually dedicate less time to oral reading, which may generate a stabilization in reading fluency, which suggest an alert for deficits in reading fluency.

The results of the present study revealed an improvement in reading fluency during e-learning. Based on this finding, we can interpret that e-learning can be a viable alternative for the assessment of reading fluency. Furlong et al. 25 also concluded, through a quick review of studies performed between 2007 and 2020, that e-learning assessments can be as effective as face-to-face assessments of the reading and spelling abilities of school-age students.

E-learning studies performed by Bao et al. 6 and Baschenis et al. 26 during the pandemic also showed an improvement in reading fluency that was lower than expected for the students. This finding was considered a reflection of the sudden and prolonged closure of schools.

It is clear, through the results of the present study, that grade 2 students suffered the most negative effects to their reading development. Specifically, the result of grade 2 in 2021 may be related to literacy during the period when schools were closed and there was no e-learning due to the pandemic. It is difficult to teach to read and write virtually, and the modality does not consider the specifics of the literacy process. 11

If we use the research conducted by Alves et al. 18 with Brazilian students as background, the results found in the present study for grades 3 and 5 in 2020 and 2021 suggest deficits in reading fluency. Table 1 shows that, in 2020, grade 5 reached an average WPM of 96.34. Table 2 shows that, in 2021, both grades 5 (2020 and 2021) had averages that also suggest a deficit (WCPM of 92.33 and 92.30 respectively), which is in line with the results found by Alves et al. 18 (WPM and WCPM between 88 and 98).

Regarding the grade 3, it is clear in Table 4 that both the classes of 2020 and 2021 also fit into the values suggested for an alert for deficits (WCPM between 59 and 71), showing WCPM averages of 70.856 and 70,830 respectively. 18

Figure 3 illustrates the development in the reading performance of the classes in 2020 and 2021, and one can see that the prepandemic classes showed better results in the rate of accuracy of reading in almost every grade. In addition, one can also see an increase in development in measurements from grades 2 to 5, reinforcing the findings of the longitudinal study and the perspective that reading ability undergoes a construction process each year and continuously evolves as the students use their writing skills. 9 22 23

Although the present study demonstrated important characteristics of the development of reading fluency in a sample of Brazilian students during the COVID-19 pandemic, the results must be carefully interpreted due to the impact of the sample size. Brazil is a country of large dimensions and important regional differences. Thus, larger studies with more representative samples would enable a more accurate understanding of the country. Also, other variables should be analyzed, such as stress, depression, anxiety in the students, cognitive profile, and home support to learn.

In conclusion, the present research showed, in the longitudinal study, that students from grades 2 to 5 developed their reading fluency during the social distancing due to COVID-19. In addition, there was an expressive development in grades 2 and 3, while the following classes showed greater stability. In the cross-sectional study, it was evident that grade 2 suffered the highest negative impact due to school closures during the pandemic, and they had poorer results in 2021 than in 2020.

The present study also highlights the need for a careful selection of the reading texts used to assess a child's reading ability.
